# Paraspinal tophi

**DOI:** 10.1136/ard-2024-226687

**Published:** 2024-09-26

**Authors:** Juan Wu, Yun Zhang

**Affiliations:** 1 Department of Family medicine & Division of General Internal Medicine, Department of Medicine, Peking Union Medical College Hospital, Beijing, China

**Keywords:** Gout, Arthritis, Low Back Pain

**Figure 1:**
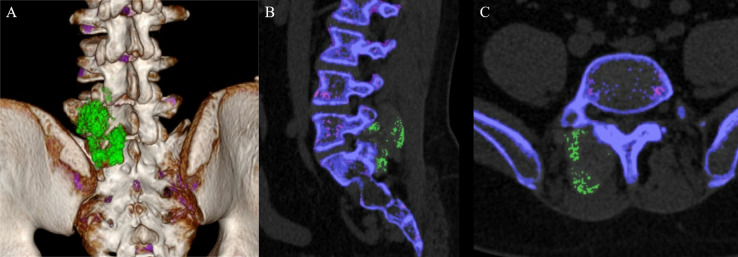
Dual-energy CT scans identified monosodium urate crystal deposits within the paraspinal region (A: coronal section; B: median sagittal section; C: transverse section).

 A young adult patient with gout was incidentally found to have a large mass on the right lumbar spine and sacrum during an abdominal CT scan. A subsequent dual-energy CT (DECT) scan confirmed this mass to be a tophus (figure 1). The patient has a history of recurrent joint swelling and pain affecting bilateral hands, wrists, elbows and left foot. Physical examination revealed the distribution of small tophi around the joints of both hands, but no subcutaneous tophus was observed. The patient had been on an irregular urate-lowering therapy since 2020. Intermittent monitoring revealed ranging serum uric acid levels from 271 to 671 µmol/L. The patient has no family history of neoplastic or autoimmune diseases.

In June 2024, a comprehensive re-evaluation revealed midline growth abnormalities resulting in central diabetes insipidus and impaired thirst regulation—an indication that the patient experienced polyuria with the absence of thirst, causing intermittent fluid volume reduction. Significant fluctuations in blood volume caused by this condition led to corresponding variations in serum creatinine and uric acid levels. Irregular urate-lowering therapy contributed to the progression of the disease. The series of escalations together resulted in the development of refractory gout. The paraspinal mass was discovered during the current hospitalisation. No symptoms such as lumbar swelling, pain or restricted movement were reported.

Performing biopsy from a deep-seated paraspinal mass in the lumbar and sacral regions surrounded by nerve roots had innate risks. To avoid potential complications, DECT was conducted over biopsy to clarify the diagnosis.

Although the effect of gout on peripheral joints has been extensively studied, axial involvement like paraspinal tophi holds insufficient recognition due to their rarity. Paraspinal tophi share similarities with infections, neoplasms and other inflammatory diseases, which can complicate diagnosis.^1^ Some patients may show symptoms such as pain in back, radiculopathy or myelopathy, while others can remain asymptomatic.^2^ Imaging modalities—DECT in particular for its advantage in specifically identifying urate crystals—play a crucial role in the diagnosis.^3^ DECT, despite its high cost and scarcity, has valuable use in the examination of axial joints, where biopsy or ultrasound evaluation is less effective. For peripheral joint or subcutaneous tophi, microscopic and ultrasonic identification are still the preferred diagnostic methods.
